# Supplementary material for: Listeria motility increases the efficiency of epithelial invasion during intestinal infection
Source: PLoS Pathog. 2022 Dec 30;18(12):e1011028. doi: 10.1371/journal.ppat.1011028 (PMC9836302; doi:10.1371/journal.ppat.1011028)
Supplement: S1 Methods — This file contains supplemental methods and includes citations [66–71]. (PDF) [file ppat.1011028.s001.pdf]

Supplementary Materials

Listeria motility increases the efficiency of epithelial invasion during intestinal infection

Inge M. N. Wortel [1,†,*], Seonyoung Kim [2,†], Annie Y. Liu [2], Enid C. Ibarra [2], and Mark J. Miller [2,*]

[†]These authors contributed equally to this work.

[*]corresponding authors
E-mail: inge.wortel@ru.nl, mmiller23@wustl.edu

[1]Data Science, Institute for Computing and Information Sciences, Radboud University, Nijmegen, the Netherlands
[2]Department of Internal Medicine, Division of Infectious Diseases, Washington University School of Medicine, St. Louis, USA

S1 Methods

Filtering for motile Lm. To filter Lm-RT "motile" tracks (Lm-RTm), each track was modelled either as (i) a single gaussian distribution of cell positions (ignoring the links between coordinates, with mean the average position and SD σ=1µm), or (ii) two gaussians of unlinked positions (one for the first m coordinates and one for the rest). The bayesian information criterion (BIC) was then computed for model (i) versus the possible models (ii) for all choices of m, and compared using ΔBIC=BIC_i-BIC_{ii}. The more positive this number gets, the more evidence there is for model ii over model i – i.e., the more evidence that the cell has moved. We then selected tracks with ΔBIC>50, an empirically determined threshold that separated clearly motile versus clearly non-motile tracks reasonably well (S2 Fig).

Estimating motility statistics. To estimate the motility coefficient M and the persistence time P of the Lm-37 and Lm-RT(-m) populations (S1 Fig), we computed mean squared displacements using *celltrackR*[1] (RRID:SCR_021021), focusing on the initial part where Δt<5sec (where MSD values are still based on many independent tracks and not biased by artefacts from cells leaving the imaging window[2]. These data were then fitted using the (2-dimensional) Fürth's equation to obtain M and P:

$$\text{MSD} = 4M[\Delta t - P(1 - e^{\Delta t/P})] \tag{1}$$

Data were fitted in R using the function nls (port algorithm, starting values [D=10, P=0.005], lower limits [D=0, P=0.001]). This process was bootstrapped to obtain a measure of uncertainty for the estimates of M and P: each of 1000 bootstrap rounds, tracks were sampled with replacement to get a new track dataset of the same size as the original, and the process above was repeated to obtain the distributions of estimated M and P.

The persistence P was also estimated from the autocovariance, using a similar procedure: autocovariances were computed in *celltrackR*, again only for the initial Δt<5sec, and fitted to an exponential decay function:

$$f(x) = Ae^{-\Delta t/P} \tag{2}$$

again using nls (port algorithm, starting values [P = 0.5, A = half of the autocovariance at Δt=1], lower bounds [A = 0.001, P = 0.01]).

CPM dynamics. For a full description of the CPM we refer the reader elsewhere[3–5], but we briefly describe the main dynamics here. At any point in time, CPM pixels p can only belong to one cell or background, but these pixel identities σ_p can change over time as follows: a randomly selected "source" pixel p_s tries to copy its identity into that of a (randomly chosen) neighboring "target" pixel p_t. If this succeeds, p_t becomes part of the cell of p_s (changing $\sigma(p_t) \rightarrow \sigma(p_s)$); otherwise nothing changes. The success rate of these copy attempts depends on their effect on the "Hamiltonian" H, an energy function defined by the modeler (see below). If the proposed change is energetically favorable (ΔH <0), it always succeeds ($P_{\text{copy}} = 1$), otherwise, its success probability becomes:

$$P_{\text{copy}} = e^{-\Delta H/T} \tag{3}$$

Every MCS, this process is repeated once for every pixel in the model. The temperature T determines how much noise (unfavorable changes) the model permits. The model behavior is thus mostly governed by the choice of $(\Delta)H$. Below, we list the most important properties and parameters for each model layer.

Epithelial CPM. The epithelium was modelled using a standard equation for ΔH, yielding a basic CPM[6] with 5 parameters. The cell volume V (an area in our 2D model), was chosen to match the typical interface of epithelial cells with the mucus/lumen. Based on images of epithelial cells (Fig S9A), we can roughly model these as circles with a radius of 8.4µm, yielding an area of ~55.4µm^2 = (2pix/µm x 55.4µm) x (2pix/µm x 55.4µm) ≈ 222 pixels. The other parameters determine epithelial dynamics: $J_{\text{cell,background}}$ = 20, $J_{\text{cell,cell}}$ = 30, λ_{volume} = 50, T = 20.

Target cell frequency in the model was estimated based on our own observations and the published work of others[7,8] that showed goblet cells represent between 4-12% of epithelial cells in the mouse small intestine. Varying target cell percentage within this range has only modest effects on model outcomes as the epithelium is relatively static compared to the phagocytes/bacteria.

Using these parameters, 13 x 13 = 289 epithelial cells were seeded, equally spaced, on the CPM. Cells were initialized as circles with a 6-pixel radius and, before the start of the simulation, allowed to equilibrate for 20 MCS to expand and cover the entire grid. N_{goblet} cells were then randomly appointed target cells representing "goblet cells" that can potentially be invaded by bacteria unless otherwise mentioned, we used N_{goblet} = 20 so that 7% of the epithelial surface is occupied by goblet cells, which is within range of experimental values[7].

Bacterial CPM. In the bacterial CPM, sLm were modelled as cells that must always occupy either one or two (neighboring) pixels (2-4µm); this roughly matches Lm size. By sequentially adding a new neighboring pixel to the cell and retracting the old pixel, these bacteria can move; but without further constraints this motion is diffusive only. To mimic the directional persistence observed in real bacteria, we used a slight modification of the Beltman et al. model[9]:

$$\Delta H = -\lambda_{\text{dir}}||\vec{a}|| \cdot ||\vec{b}|| \cos\alpha = -\lambda_{\text{dir}}||\vec{a}|| \cdot \cos\alpha \tag{4}$$

Here, $\vec{b}$ is the normalized displacement vector of the bacterium over the last Δt steps (because of the normalization, its length $||\vec{b}||$ = 1). $\vec{a}$ is the vector pointing from the source pixel p_s to the target p_t; unlike in Beltman et al.[9] it is not normalized, reflecting the slightly larger distance of copy attempts over the diagonal compared to copy attempts in vertical/horizontal directions ($||\vec{a}|| = \sqrt{2}$ pixels in the former case and 1 pixel in the latter). α is the angle between $\vec{a}$ and $\vec{b}$, its cosine is +1 when the copy attempt perfectly aligns with the previous cell direction, and -1 when the two are polar opposites. Thus, copy attempts in the direction of previous movement are favored with a negative ΔH whose magnitude depends on the parameter λ_{dir}.

This model yields motility that roughly matches the persistent random walks exhibited by motile bacteria. The most important parameters controlling this motion are Δt (controlling the persistence time over which the previous cell direction is taken into account) and λ_{dir} (containing the strength of this directionality). Because Lm move at much higher speeds than phagocytes do, the temporal resolution of the bacterial CPM was set higher than that of the rest of the model: for every MCS of the main model, v_{rel} steps of the bacterial model are performed. Thus, v_{rel} is another model parameter scaling bacterial speed relative to other processes. Δt, λ_{dir}, and v_{rel} were fitted to match in vitro Lm motility; see "Lm motility parameters" below.

At the beginning of each simulation, N_{bac} bacteria were randomly placed in the field with random initial directions. Except in simulations where challenge dose was varied, we used N_{bac} = 100.

Lm motility parameters. To model Lm-RT motility,λ_{dir}, Δt and v_{rel} were chosen to match motility statistics of in vitro Lm-RT-m motility data. Because some motility measurements are influenced by imaging window size[2], a separate bacterial layer simulation was set up to match the imaging window of the Lm motility data (348.5 x 261.4µm = 697 x 523 pixels), with periodic borders. 50 bacteria were simulated for 30 x v_{rel} steps (=30sec) at a given set of parameters, recording centroids every 10 steps. Simulated tracks were then (1) split whenever cells crossed the periodic boundary, to mimic cells entering and leaving the field of view, and (2) interpolated to the same framerate as the original data (every 0.25sec). T was (arbitrarily) fixed to 20. λ_{dir}, Δt and v_{rel} were tuned manually until simulated cells had similar speed distributions, MSD, and autocovariance as vitro Lm-RT (computed in *celltrackR*[1], RRID:SCR_021021, (S5 Fig).This yielded λ_{dir} = 40, v_{rel} = 150 steps/sec, and Δt=60 steps (on the bacterial CPM, which is 60/v_{rel} = 60/150 = 0.4sec in real time). To model non-motile Lm-37, λ_{dir} was set to 0 (ensuring that ΔH=0 regardless of Δt; removing all directional persistence). v_{rel} was set to 1 to allow Lm-37 some slight diffusive motion, but nowhere near as fast as Lm-RT.

Phagocyte CPM. Phagocytes were modelled using the Act-CPM, which has been shown to capture realistic mammalian cell shapes and motility in the CPM[10,11]. For details we refer to these publications. The cell volume V was set to 314 pixels, corresponding to the 78.5µm^2 area obtained for cells with a diameter of ~10µm (roughly the scale of the cells in the neutrophil imaging data). The perimeter P was set to 230 to get a reasonable circumference for that cell size. Most other parameters were chosen as in Niculescu et al.[11]: $J_{\text{cell,background}}$ = 20, $J_{\text{cell,cell}} = 100$, λ_{volume} = 50, T = 20. The parameter $\lambda_{\text{perimeter}}$ was set to 1.5, slightly lower than in Niculescu et al.[11] to make cells slightly more deformable. All these parameters together determine cell size, morphology, and deformability. Migratory behavior in this model is predominantly governed by two additional parameters, max_{act} and λ_{act}, which were tuned as described below.

Phagocyte motility parameters. The phagocyte motility parameters max_{act} and λ_{act} were fitted to neutrophil motility data in a similar manner as described in "Lm motility parameters" above, now using a simulation of 300 x 400 pixels (matching the 150 x 200µm imaging window) with 40 phagocytes simulated for 1000 MCS (16min). Centroids were recorded every 5s and tracks postprocessed as described above for the Lm motility data. max_{act} and λ_{act} were tuned to match speed distributions, MSD, and autocovariance (S10 Fig),yielding max_{act} = 20 and λ_{act} = 400. In simulations with "non-motile" phagocytes, both parameters were set to 0.

Invasion & phagocytosis dynamics. After every step in the bacterial CPM (i.e. v_{rel} times per second), each bacterium was allowed to interact with the epithelium and phagocytes as follows:

1. If the bacterial pixel(s) fully overlapped a phagocyte on the phagocyte layer, it could be phagocytosed with probability $p_\phi = k_\phi / v_{\text{rel}}$; if successful, the bacterium was removed.
2. If the pixel(s) of a remaining, motile bacterium fully overlapped with a target cell on the epithelial layer, it could attach with a probability $p_{\text{attach}} = k_{\text{attach}} / v_{\text{rel}}$. Such bacteria can no longer leave the target cell they are attached to, but can still be phagocytosed as in (1), or can invade as in (3) below.
3. Any attached bacteria can fully invade with probability $p_{\text{infect}} = k_{\text{infect}} / v_{\text{rel}}$". Once invaded, bacteria can no longer be phagocytosed. They remain in the simulation for visualization purposes but no longer affect the behavior/dynamics of any other cells.

We here assumed that attached bacteria rapidly become intracellular based on studies of endocytosis[12,13]. We chose 5s as a starting value for initial bacteria-target cell interactions after which, bacteria become inaccessible to phagocytes during transcytosis across the epithelium. Thus, we set the average infection rate k_{infect} = 0.2 s^{-1}. We consider the first step of attachment to be the slower, rate-limiting step with a rate k_{attach} = 0.051 s^{-1}, consistent with kinetics from S4-S7 Movies in which Lm engaged with encountered goblets (rather than just moving past them) in about 10% of cases. The speed of phagocytosis depends on many factors but typically occurs in seconds rather than minutes[14,15]. Because no published data exist for neutrophil phagocytosis of Lm to set the phagocytosis efficiency k_ϕ in the model, we initially set $k_\phi = k_{\text{attach}}$ = 0.051 s^{-1} for simplicity, but later investigated the sensitivity of our conclusions to both limiting rates k_ϕ and k_{attach}.

References

[1] Wortel IM, Liu AY, Dannenberg K, Berry JC, Miller MJ, Textor J. CelltrackR: an R package for fast and flexible analysis of immune cell migration data. ImmunoInformatics. 2021;1:100003.

[2] Beltman JB, Marée AF, De Boer RJ. Analysing immune cell migration. Nature Reviews Immunology. 2009;9(11):789-98.

[3] Marée AF, Grieneisen VA, Hogeweg P. The Cellular Potts Model and biophysical properties of cells, tissues and morphogenesis. In: Single-cell-based models in biology and medicine. Springer; 2007. p. 107-36.

[4] Szabó A, Merks RM. Cellular Potts modeling of tumor growth, tumor invasion, and tumor evolution. Frontiers in oncology. 2013;3:87.

[5] Hirashima T, Rens EG, Merks RM. Cellular Potts modeling of complex multicellular behaviors in tissue morphogenesis. Development, growth & differentiation. 2017;59(5):329-39.

[6] Graner F, Glazier JA. Simulation of biological cell sorting using a two-dimensional extended Potts model. Physical review letters. 1992;69(13):2013.

[7] Kaji I, Roland JT, Rathan-Kumar S, Engevik AC, Burman A, Goldstein AE, et al. Cell differentiation is disrupted by MYO5B loss through Wnt/Notch imbalance. JCI insight. 2021;6(16).

[8] D'Angelo A, Bluteau O, Garcia-Gonzalez MA, Gresh L, Doyen A, Garbay S, et al. Hepatocyte nuclear factor 1α and β control terminal differentiation and cell fate commitment in the gut epithelium. Development. 2010;137(9):1573-82.

[9] Beltman JB, Marée AF, Lynch JN, Miller MJ, De Boer RJ. Lymph node topology dictates T cell migration behavior. The Journal of experimental medicine. 2007;204(4):771-80.

[10] Wortel IMN, Niculescu I, Kolijn PM, Gov NS, de Boer RJ, Textor J. Local actin dynamics couple speed and persistence in a cellular Potts model of cell migration. Biophysical journal. 2021;120(13):2609-22.

[11] Niculescu I, Textor J, De Boer RJ. Crawling and gliding: a computational model for shape-driven cell migration. PLoS computational biology. 2015;11(10):e1004280.

[12] Pelassa I, Zhao C, Pasche M, Odermatt B, Lagnado L. Synaptic vesicles are "primed" for fast clathrin-mediated endocytosis at the ribbon synapse. Frontiers in molecular neuroscience. 2014;7:91.

[13] Smith SM, Renden R, von Gersdorff H. Synaptic vesicle endocytosis: fast and slow modes of membrane retrieval. Trends in neurosciences. 2008;31(11):559-68.

[14] Lin A, Loughman JA, Zinselmeyer BH, Miller MJ, Caparon MG. Streptolysin S inhibits neutrophil recruitment during the early stages of Streptococcus pyogenes infection. Infection and immunity. 2009;77(11):5190-201.

[15] Heinrich V. Controlled one-on-one encounters between immune cells and microbes reveal mechanisms of phagocytosis. Biophysical journal. 2015;109(3):469-76.

Supplemental Table

Table S1: Overview of model parameters. For details on how parameters were selected, see the Supplemental methods. Most important parameters are highlighted with •.

Component	Parameter		Description	Value (baseline)	Further remarks
GENERAL	Time resolution		# steps of the main model run for every second in real time	1 MCS/sec	-
	Spatial resolution		# pixels per µm	2 pixels/µm	-
	Runtime		duration of the simulation	3600 MCS (1 hour)	-
EPITHELIAL CPM	V		# pixels per cell	222 pixels	-
	λ_{volume}		Unitless scaling of "elasticity" of the cell size	50	-
	$J_{\text{cell,background}}$		Surface tension cell to background	20	-
	$J_{\text{cell,cell}}$		Surface tension between epithelial cells	30	-
	T		"temperature" controlling stochasticity	20	-
	N_{epi}		Number of epithelial cells	13 x 13 = 289	-
	N_{goblet}	•	Number of goblet cells within N_{epi}	20	Varied in S11 Fig
BACTERIAL CPM	λ_{dir}	•	Unitless scaling of how strongly a cell "persists" in its direction of motion	40 for Lm-RT, 0 for Lm-37	Set to match Lm motility; see S5 Fig
	Δt	•	Duration for which cells remember their previous direction, sort of "persistence time"	60 steps in the bacterial model	$60/v_{\text{rel}} = 60/150 = 0.4$s in real time. Set to match Lm motility; see S5 Fig
	v_{rel}	•	# steps of the bacterial CPM run for every second in real time	150 for Lm-RT, 0 for Lm-37	Set to match Lm motility; see see S5 Fig. Varied in S6 Fig, S13 Fig
	T		"temperature" controlling stochasticity	20	-
	N_{bac}	•	# bacteria	100	Varied in S11 Fig
PHAGOCYTE CPM	V		# pixels per cell	314 pixels	-
	P		Cell perimeter	230	-
	λ_{volume}		Unitless scaling of "elasticity" of cell size	50	-
	$\lambda_{\text{perimeter}}$		Unitless scaling of "elasticity" of perimeter	1.5	-
	$J_{\text{cell,background}}$		Surface tension cell to background	20	-
	$J_{\text{cell,cell}}$		Surface tension between phagocytes	100	-
	λ_{act}	•	Unitless scaling of protrusion "strength"	400 (0 for non-motile)	Set to match neutrophil motility; see S10 Fig
	$\max_{\text{act}}$	•	Protrusion "memory" in MCS	20 (0 for non-motile)	Set to match neutrophil motility; see S10 Fig
	N_{ph}	•	# phagocytes	14	~ matching the total area of goblet cells; varied in S11 Fig
LAYER INTERACTIONS	k_{attach}	•	Rate by which motile bacteria attach to target cells	0.051 sec^{-1}	Varied in S7 Fig, S12 Fig
	k_{infect}	•	Rate by which attached bacteria reach the "point of no return" when invading	0.2 sec^{-1}	Has a relatively small effect when k_{attach} is rate-limiting
	k_{ϕ}	•	Rate by which bacteria (free/attached) get phagocytosed by overlapping phagocytes	0.051 sec^{-1}	Varied in S12 Fig
