# Supplementary figures and images for: Listeria motility increases the efficiency of epithelial invasion during intestinal infection
Source: PLoS Pathog. 2022 Dec 30;18(12):e1011028. doi: 10.1371/journal.ppat.1011028 (PMC9836302; doi:10.1371/journal.ppat.1011028)

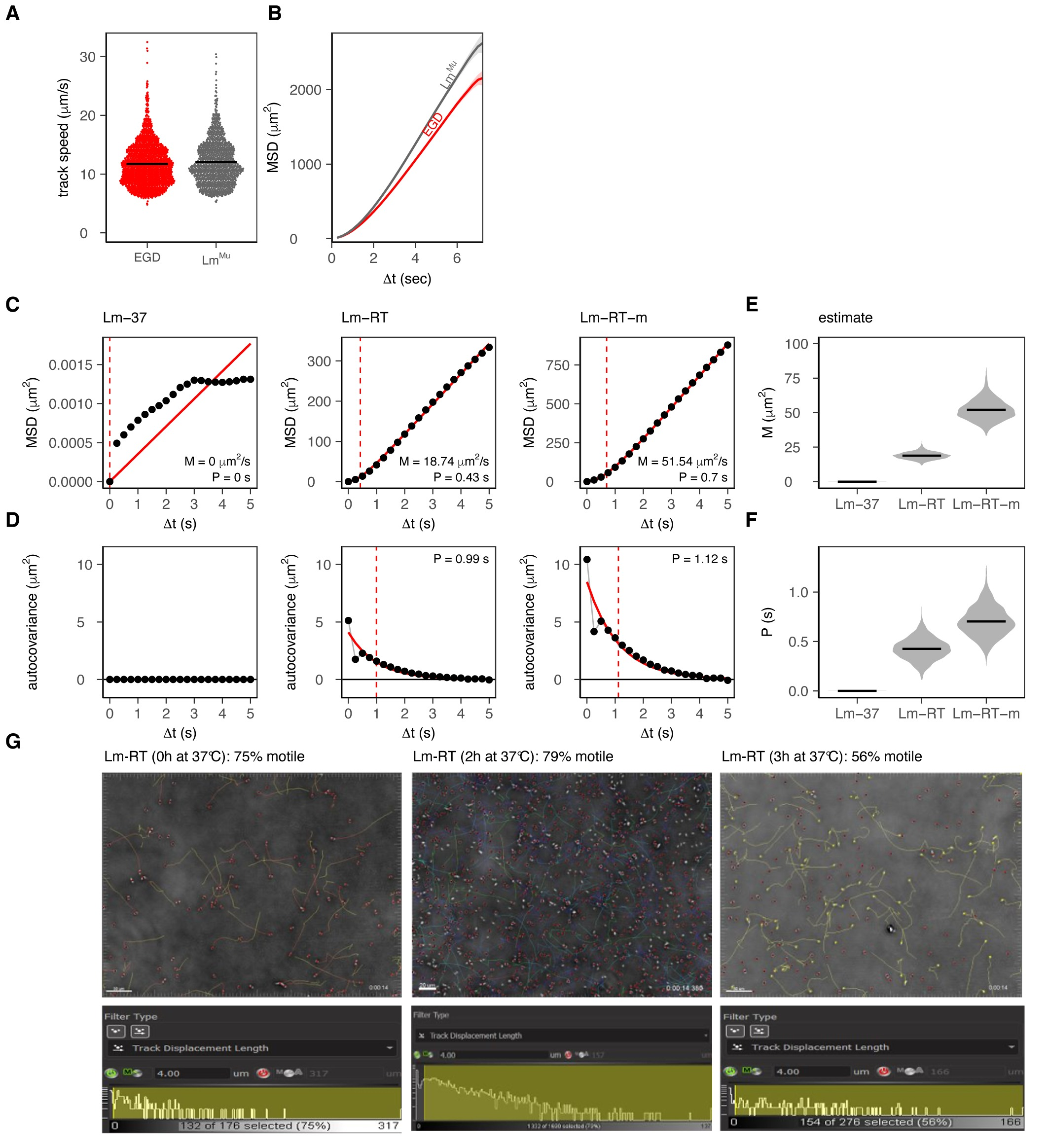

Supplement: S1 Fig — A,B: Comparison of speed (A) and mean squared displacement (MSD, B) between human EGD Lm and murinized LmMu. C: MSD curve of the three Listeria populations (EGD Lm-37, Lm-RT, Lm-RT-m), fitted by Fürth’s equation (red line). The red dashed line indicates the persistence time as determined from the fit. Curves were fit on Δt up to 5s; for longer Δt, fast cells tend to leave the imaging window and the MSD becomes biased (see Methods for details). Lm-37 did not move and could not be fitted by Fürth’s equation. D: Autocovariance curve of the populations as fitted by an exponential decay: f(x) = f0*exp(−x/P), red line. This yields a slightly higher estimate of the persistence time P (vertical dashed red lines), but still in the same order of magnitude as those in panel C. E,F: To estimate uncertainty in motility parameters estimated from the MSD (motility coefficient M and persistence time P), tracks were resampled from the original populations with replacement N = 1000 times, to obtain N “bootstrapped” datasets of equal size as the original. Resampled datasets were then fitted with Fürth’s equation as shown in panel A to obtain N estimates of M and P. G: To assess how long Lm-RT stay motile after being placed at 37°C, Lm-InlA was first grown at RT with shaking (200rpm) to OD600 around 1.0, and then switched to a 37°C incubator (shaking at 200rpm) to assess motility after 2–3 hours. Samples were diluted 1:10 with BHI and plated on a non-charged slide (Globe Scientific Cat No 1324W); a 2D time lapse video was recorded for 15 seconds with 250ms time and 100ms exposure using Olympus IX51 inverted microscope with 20X objective and phase dichroic filter. Cells were were tracked in Imaris 9.3 and % motile cells were calculated using 4 μm track displacement length filter. (TIF) [file ppat.1011028.s019.tif]

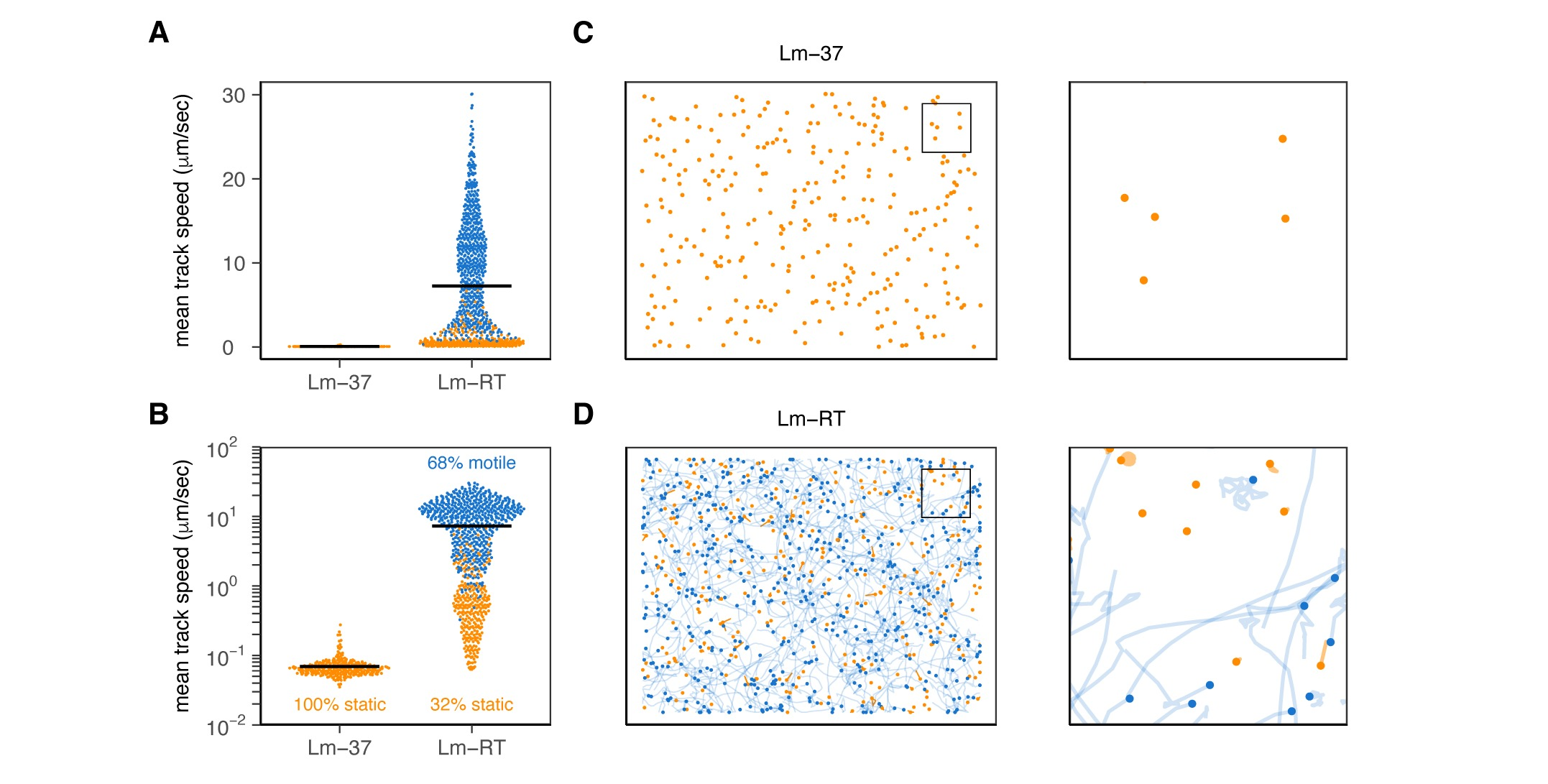

Supplement: S2 Fig — To remove artefacts of non-motile cells sticking to the glass slide, bacteria separate tracks were separated into “motile” vs “static” tracks. Briefly, tracks were considered motile whenever the track coordinates were described better by two Gaussian distributions (splitting the track in two parts) than by a single Gaussian. If a single Gaussian distribution was a reasonably good fit for the observed coordinate, the tracks were classified as static. See S1 Methods for details. A,B: static (orange) and motile (blue) cells of Lm-37 and Lm-RT, shown in the speed distribution. Whereas static tracks tend to have low speeds, there are also some static tracks with relatively high speeds (mostly when the track contains a single motile step while the cell otherwise does not move). Blue tracks represent the “Lm-RT-m” population. C,D: tracks of Lm-37 and Lm-RT, showing that the filter indeed reasonably removes non-motile cells. Zoomed inset: 50 x 50 μm. (TIF) [file ppat.1011028.s020.tif]

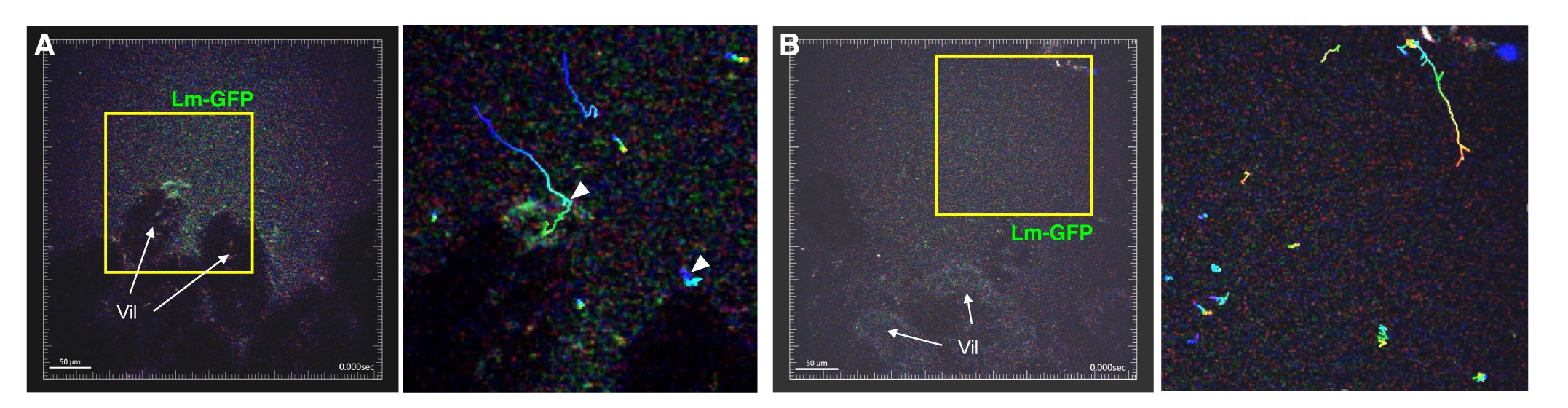

Supplement: S3 Fig — C57BL/6 mice were infected by gavage with 2x10^8^ EGD-GFP Lm, which bind poorly to mouse E-cadherin and thus allows motility to be assessed in the absence of goblet cell recognition and invasion. Mice were sacrificed 1–1.5hpi and the ileum was imaged using 2P microscopy. A,B: show motility behaviors in two different regions; in A, Lm was imaged near the villi (white arrows)as well as in the mucus and fluid phases, with tracks exhibiting both short and long persistence. The zoomed insets show time-encoded tracks of motile Lm over 12 seconds. Examples of epithelial scanning can be observed in the zoomed panel from A. Scale bar: 50μm. (TIF) [file ppat.1011028.s021.tif]

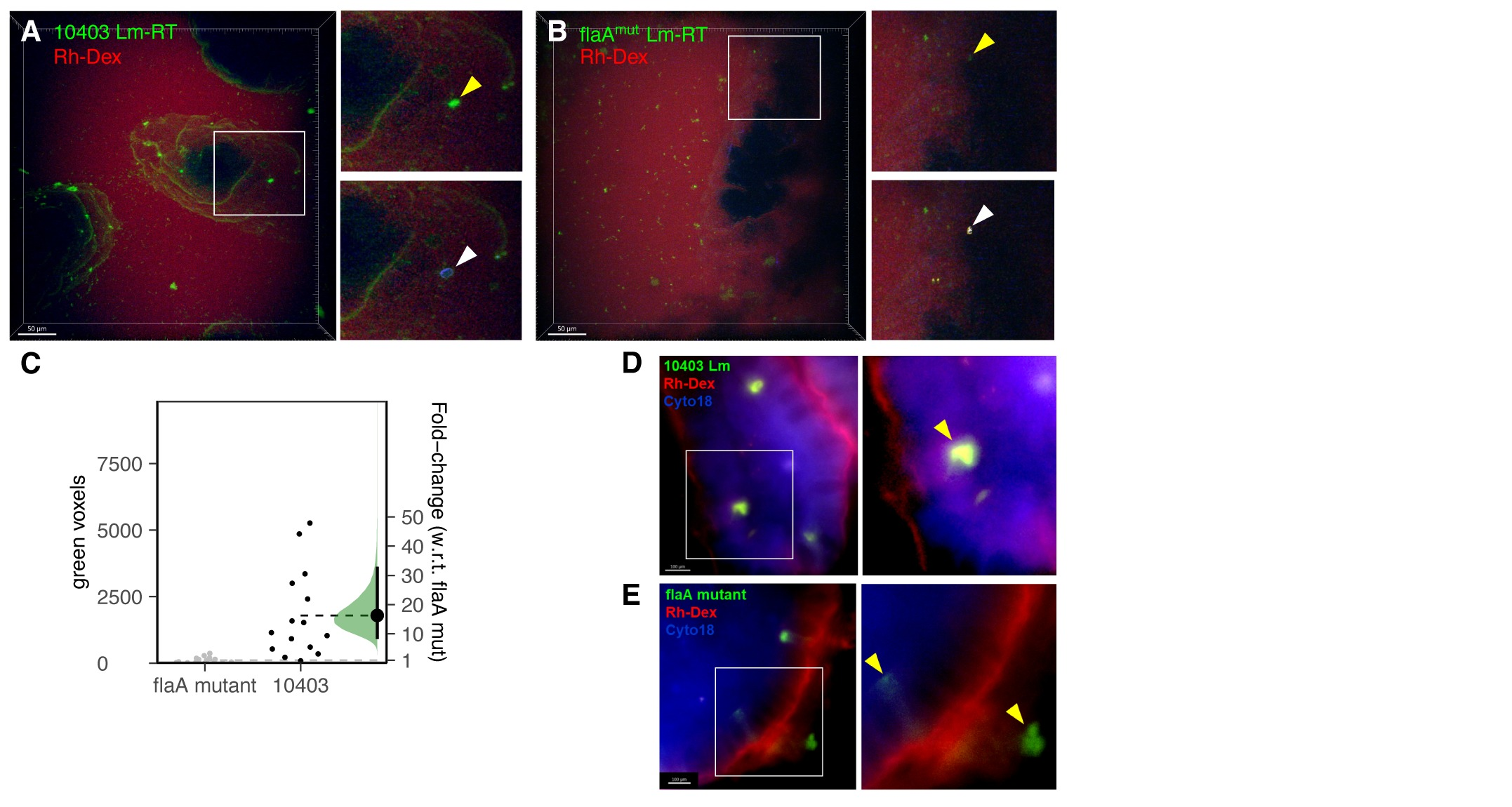

Supplement: S4 Fig — Human ileal biopsy tissue was explanted and infected with 1x10^8^ Lm-RT 10403 or flaA deletion mutant Lm. Epithelial interactions were assessed by 2P video microscopy using 10kD Rh-dextan to visualize the lumen (red). A,B: Example images for 10403 Lm (A) and the flaA mutant strain (B). Zoomed insets show examples of Lm (yellow arrows) at the epithelial surface (white arrows). Dark oval areas on the red background are villi. Scale bar: 50 μm. C: Quantification of Lm (green voxels) overlapping the epithelial surface in both strains, showing that at RT, flaA mutant Lm is deficient in reaching the epithelial surface (similar to Lm-37; see also Figs 2H, 3J and 3K). 10403 Lm overlapped with the epithelial surface about 16 times more than flaA mutant Lm did (95% CI: [8.3–33]). Each point represents an image, from a total of 2 mice per condition. D: 10403 Lm (green, yellow arrows) co-localized near large epithelial cells that were both Rh-dextran and Cytokeratin-18 staining, consistent with goblet cells. E: Lm flaA deletion mutant colocalization was comparably less (dim green, yellow arrow) and Lm often aggregated in clumps in the mucus layer (bright green, yellow arrow). In D,E, Scale bar: 100 μm. (TIF) [file ppat.1011028.s022.tif]

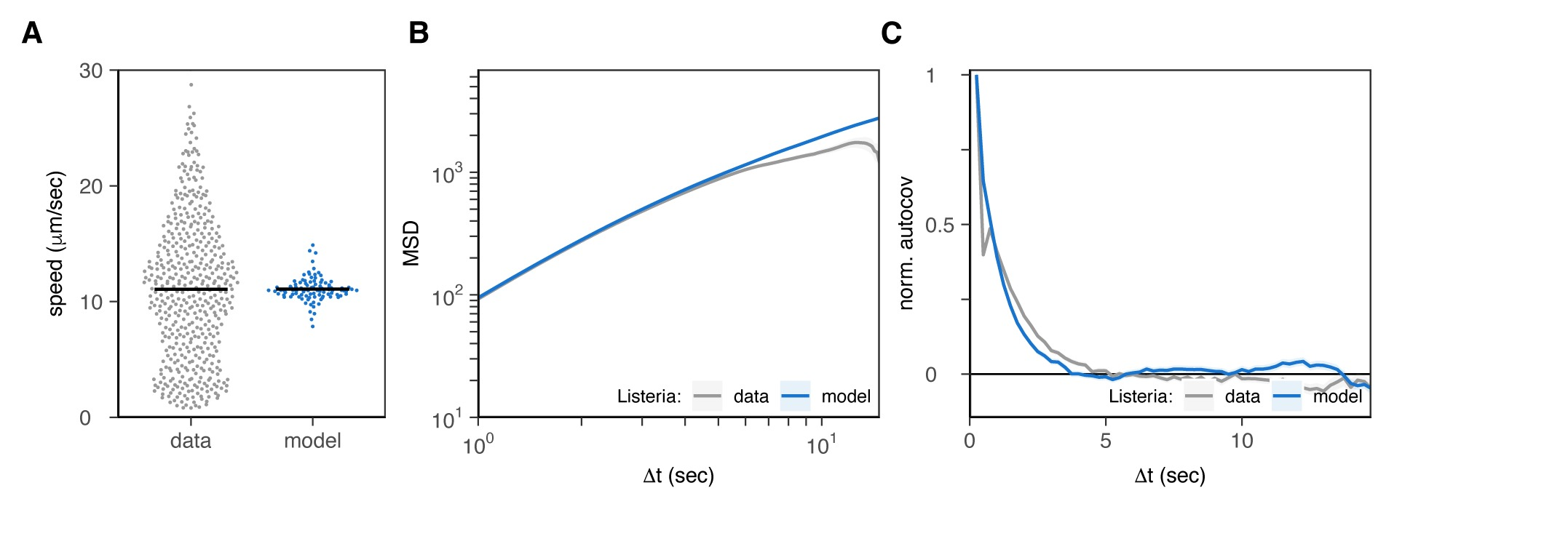

Supplement: S5 Fig — Average bacterial motility (of 50 simulated bacteria) in the model (sLm-RT) closely matches in vitro motility (Lm-RT) in both speed and directionality, as shown by: A: the distribution of cell speeds, B: the mean squared displacement (MSD) over different time intervals Δt and C: the (normalized) autocovariance of movement “step” vectors with time Δt between them (the longer it takes for this curve to drop to zero, the larger the persistence time of the cells). See Methods for details. (TIF) [file ppat.1011028.s023.tif]

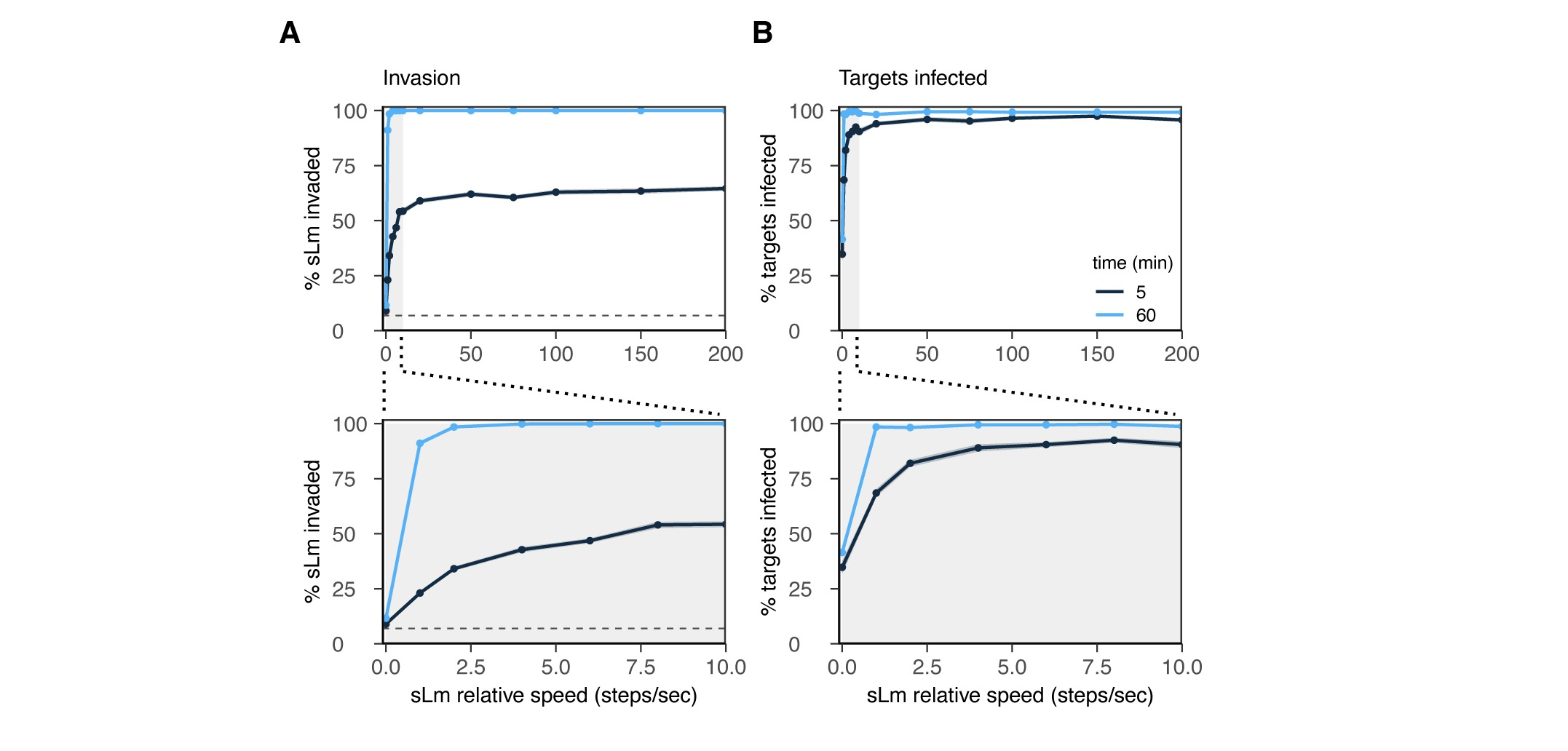

Supplement: S6 Fig — sLm speed was changed by varying the vrel parameter in the model. This parameter controls how many “steps” of the bacterial model occur each second; high values increase bacterial speed whereas a value of zero means that bacteria are completely static (the default value used throughout the paper is 150 to simulate sLm-RT and 1 to simulate sLm-37). Target cell infection efficiency is measured as A: the % of sLm that have invaded after 60 min for all tested speeds (top) or for sLm speeds up to 10 steps/s (bottom; corresponding to the gray region in the upper plot); and B: likewise, but now for the % of target cells that has been invaded by sLm. Results are shown as mean ± SE for 20 independent simulations. Horizontal dashed lines in A represent the % of the surface area covered with target cells. (TIF) [file ppat.1011028.s024.tif]

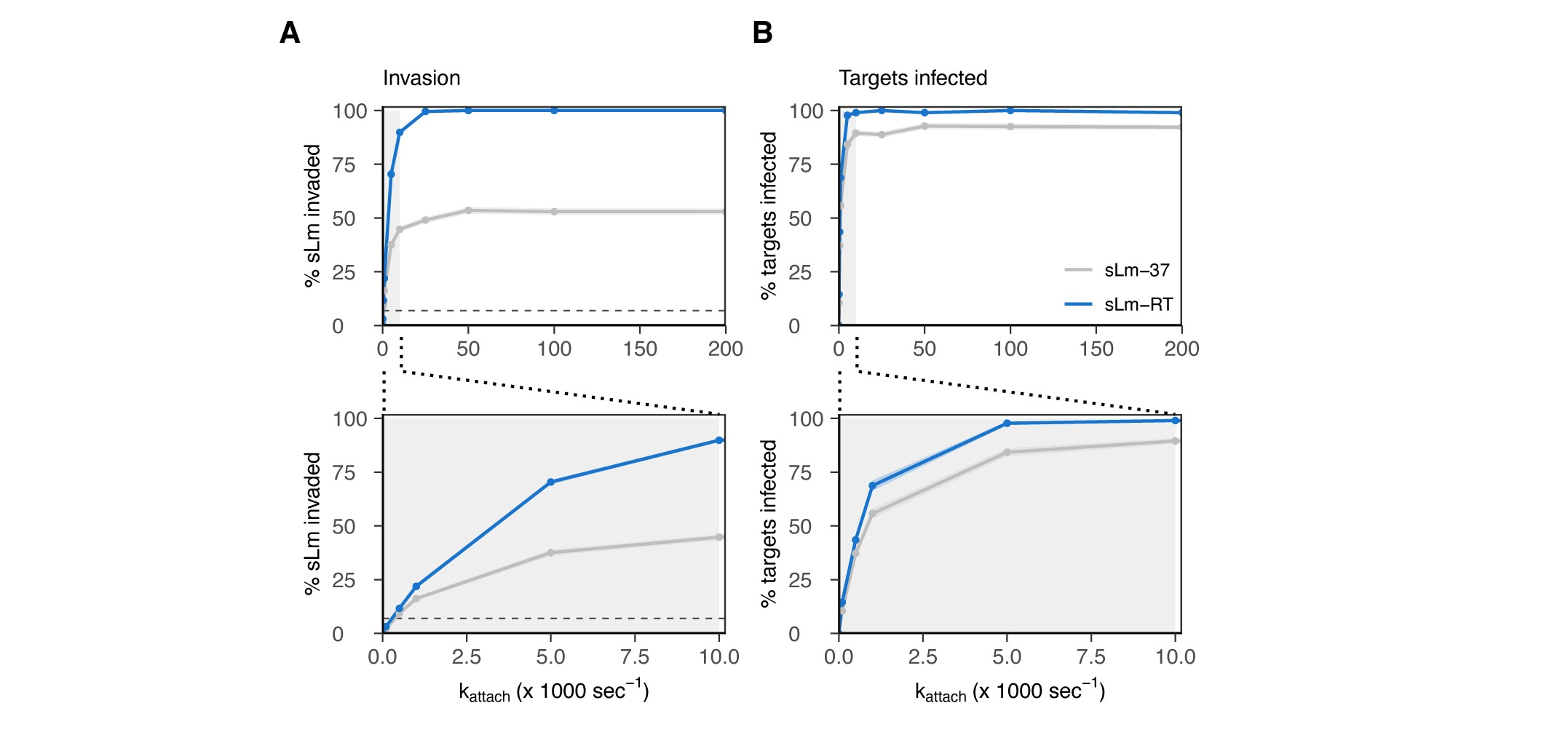

Supplement: S7 Fig — When kattach is high, scanning the epithelium will immediately attach to any target cell they encounter; when it is low, sLm are more likely to move past target cells instead of attaching to them (default value used in the paper: 0.051 s-1). Target infection efficiency is measured as A: the % of sLm that has invaded after 60 min for all tested speeds (top) or for sLm speeds up to 10 steps/s (bottom; corresponding to the gray region in the upper plot); and B: likewise, but now for the % of target cells that has been invaded by sLm. Results are shown as mea ± SE for 20 independent simulations. (TIF) [file ppat.1011028.s025.tif]

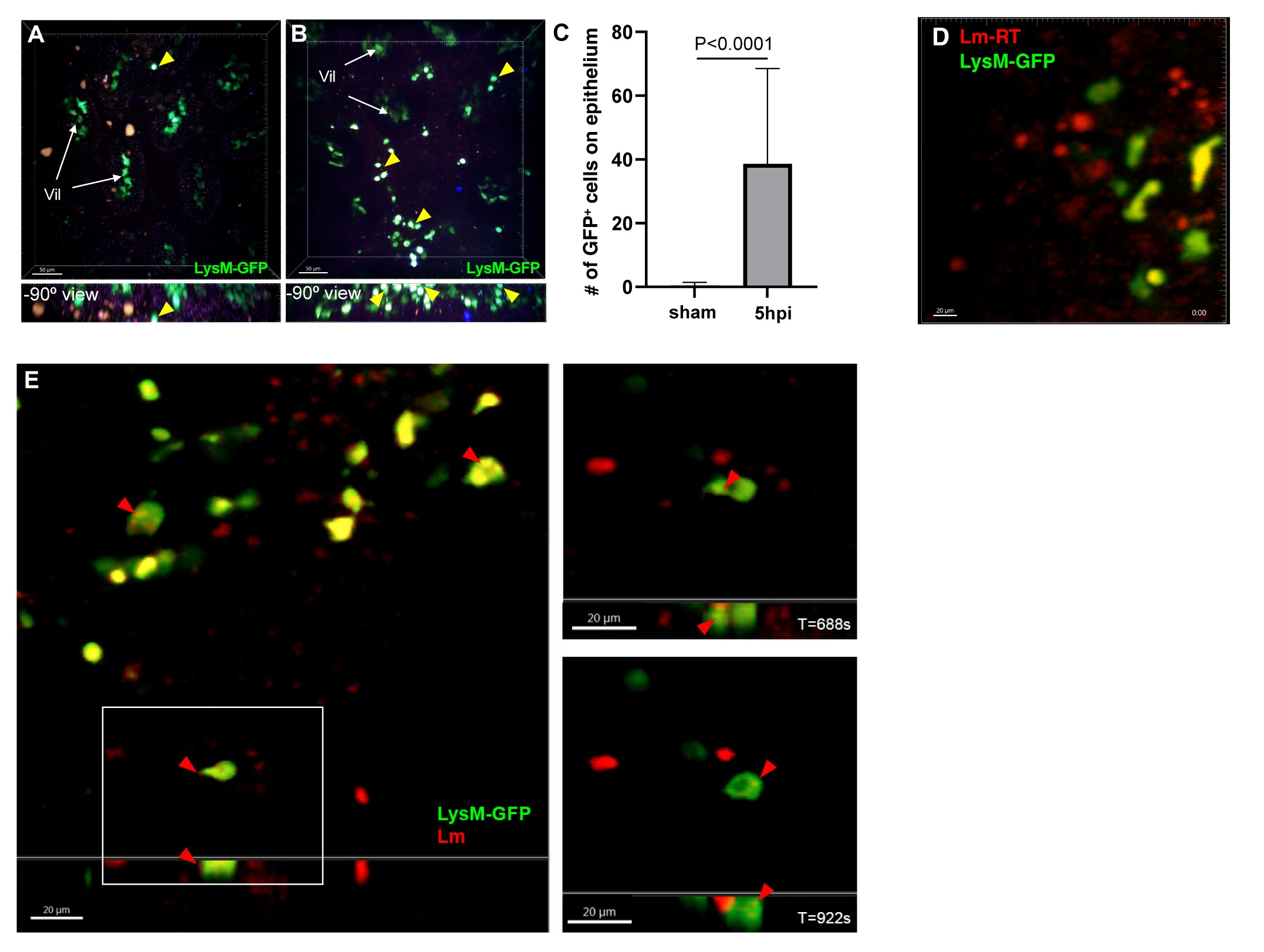

Supplement: S8 Fig — A,B: LysM-GFP mice were treated intraluminally with either vehicle (sham) or 2x10^8^ Lm. Mice were sacrificed, the ileum explanted, and 3D images collected from the luminal side to assess neutrophil (LysM-GFP) recruitment to the surface of the epithelium. Scale bar: 50 μm. C: GFP cell numbers on the epithelium (examples shown by yellow arrows), were enumerated using the spot function in Imaris and compared using a two-tailed Mann-Whitney non-parametric test. Data are from 12 (sham) and 16 (Lm-RT) images from 3 independent mice. D: Upon rechallenge, transmigrated neutrophils interact with incoming Lm at the epithelium. LysM-GFP mice were treated intraluminally with 1x10^8^ Lm. Mice were sacrificed, the ileum explanted, rechallenged with 1x10^8^ Lm (BacLight-Red labelled) and 2P imaged alongside neutrophils (LysM-GFP)at the epithelium. Scale bar: 20 μm. E: Example of Lm (red, red arrows) phagocytosis by LysM-GFP neutrophils (green). Left: overview with three examples of phagocytosis (scale bar: 25 μm), including a -90° view. Right: zoomed view for the highlighted example at two different time points (scale bar: 20μm). (TIF) [file ppat.1011028.s026.tif]

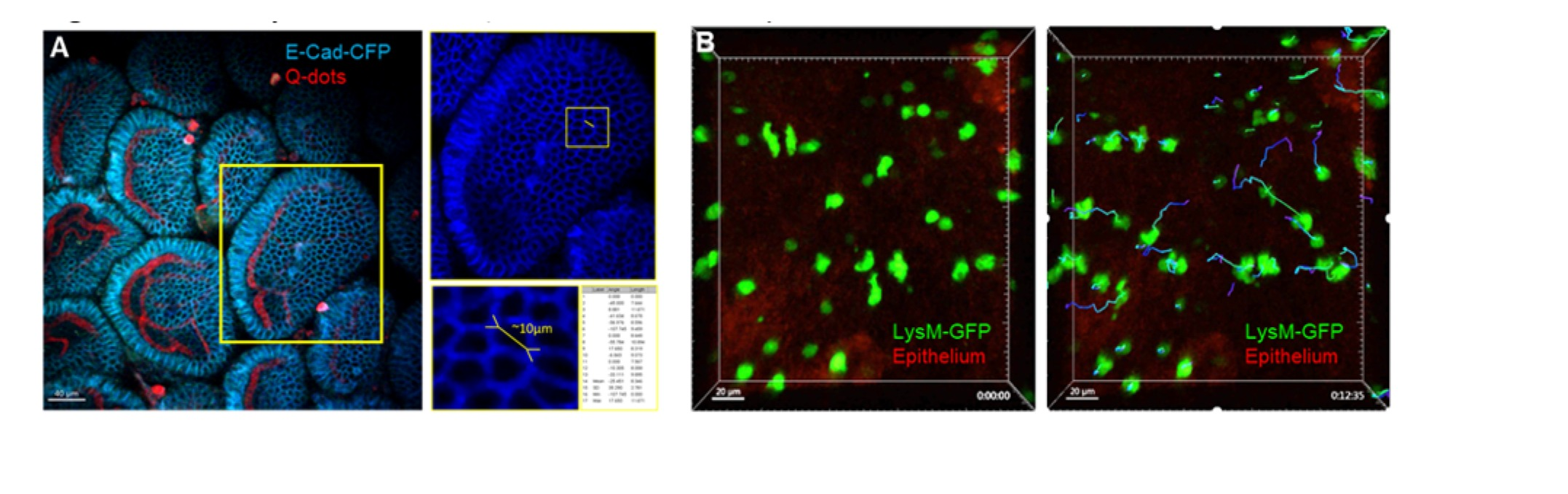

Supplement: S9 Fig — Mice were anesthetized, the ileum glued to a plastic support and carefully dissected to expose the luminal surface for 2P microscopy. A: Blood vessels were labeled with 655nm Q-dots and images of the epithelium acquired and analyzed to estimate epithelial cell dimensions and structure for the L-CPM. B: LysM-GFP mice were imaged with time-lapse 2P microscopy to assess neutrophil (green) migration dynamics on the surface of the epithelium (red). Multidimensional datasets were rendered, and cells tracked in Imaris. Tracks are time encoded. Scale bar = 20μm. Time stamp is min:sec. Neutrophil motility parameters were calculated using celltrackR/MotilityLab (2Ptrack.net) and used as the basis for phagocyte motility in the L-CPM model. (TIF) [file ppat.1011028.s027.tif]

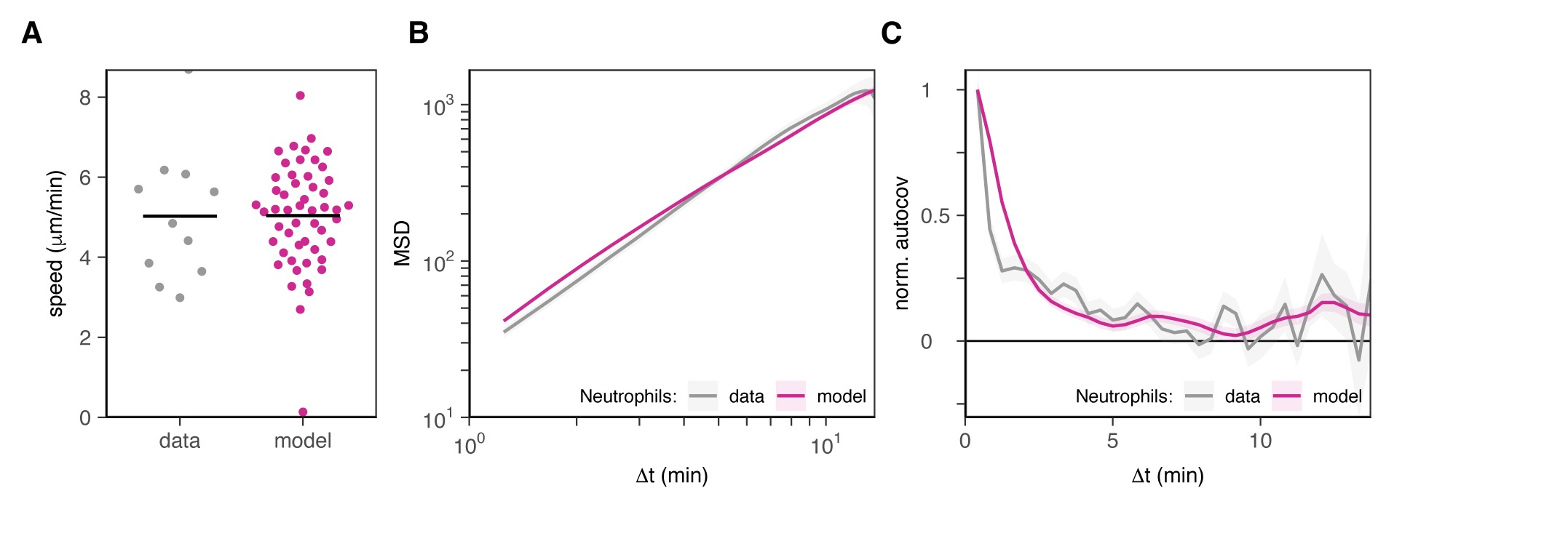

Supplement: S10 Fig — Phagocyte motility in the model (average of 40 simulated cells) closely matches in vivo motility of neutrophils crawling between epithelium and coverglass. Motility matches in both speed and directionality, as shown by: A, the distribution of cell speeds, B, the mean squared displacement (MSD) over different time intervals Δt and C, the (normalized) autocovariance of movement “step” vectors with time Δt between them (the longer it takes for this curve to drop to zero, the larger the persistence time of the cells). See Methods for details. (TIF) [file ppat.1011028.s028.tif]

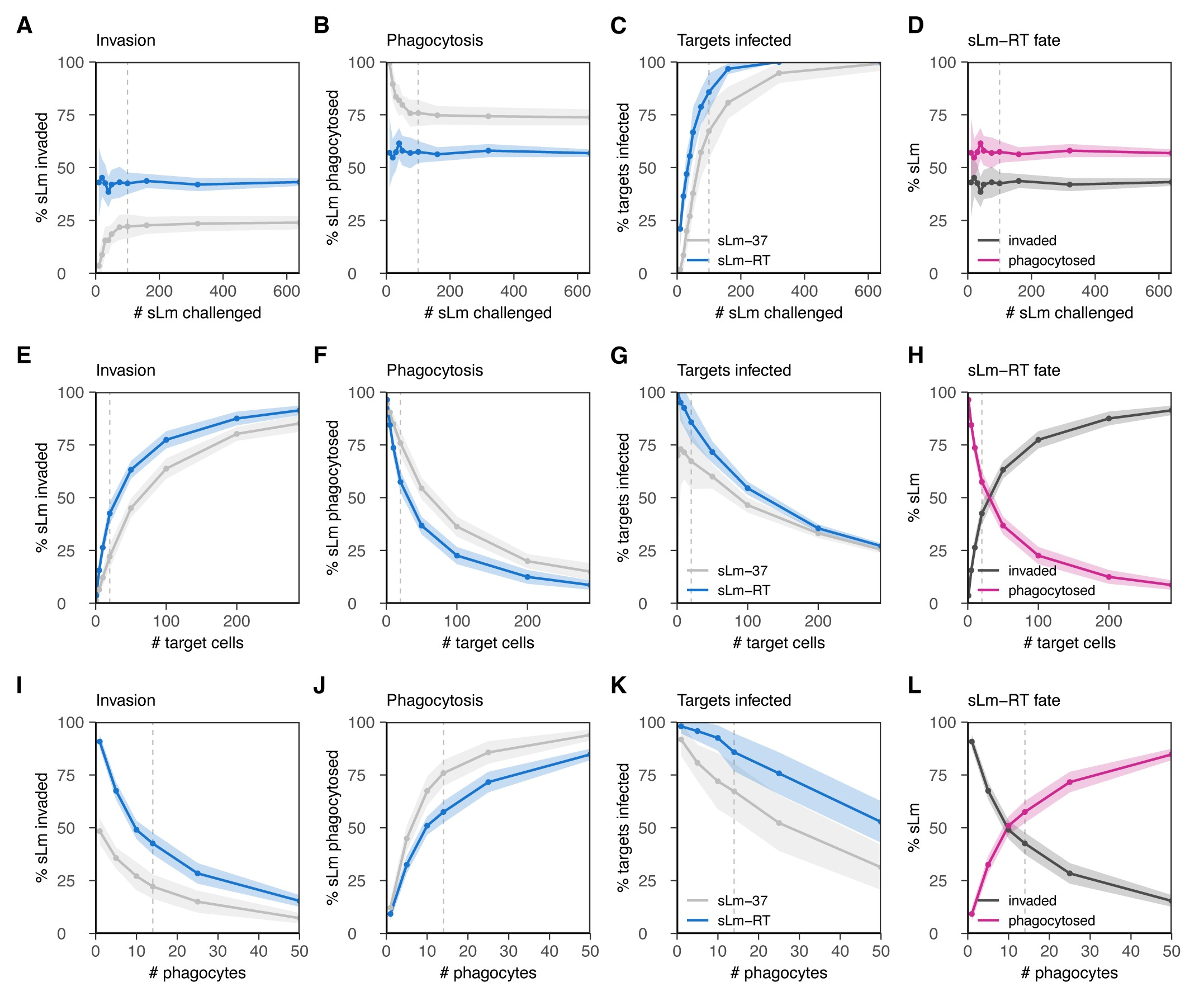

Supplement: S11 Fig — Immunological outcomes were assessed after varying (A-D): the number of sLm challenged (with A: % sLm invaded, B: % sLm phagocytosed, C: % of target cells invaded by sLm after 60 min, and D: the data from panels A-B compared in one plot for motile sLm-RT). While challenge dose does not affect the % of sLm invading or phagocytosed for motile sLm-RT (blue), it does for low doses of the non-motile sLm-37 (gray), which are more likely to diffuse on or near target cells before being phagocytosed. Dependency of immunological outcomes was similarly tested for E-H: the number of “target” cells (i.e. goblets that can be invaded; the total number of epithelial cells is 289), and I-L: the number of phagocytes. Results are shown as mean ± standard deviation (SD) for 20 independent simulations for motile (sLm-RT, blue) and non-motile bacteria (sLm-37, gray). Vertical dotted lines indicate the baseline of 100 sLm, 20 target cells, and 14 phagocytes used in the rest of the paper. (TIF) [file ppat.1011028.s029.tif]

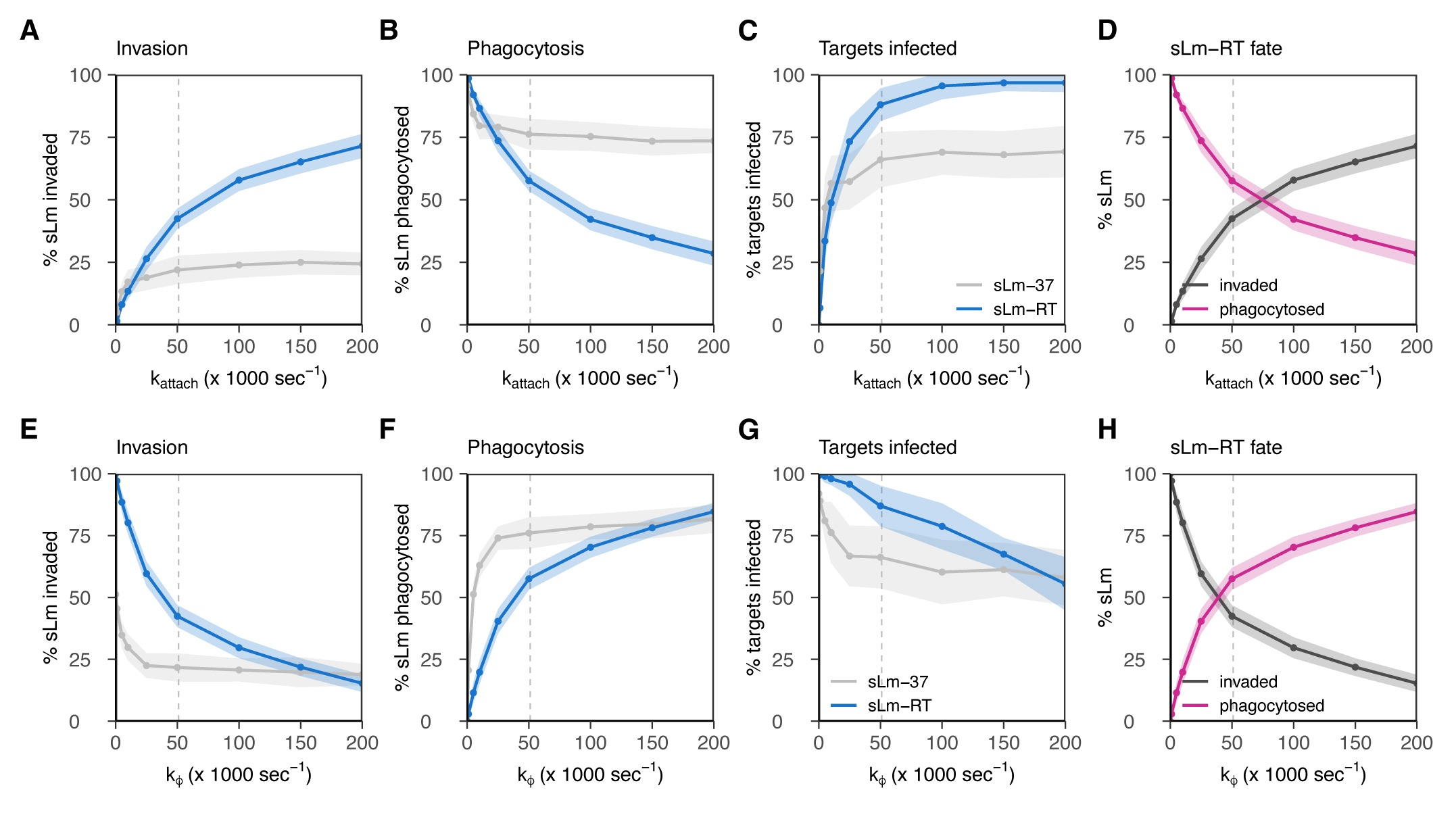

Supplement: S12 Fig — Immunological outcomes were assessed after varying (A-D): the rate by which sLm attach to target cells they encounter (kattach) or (E-G): the rate by which sLm are phagocytosed by encountered phagocytes (kφ). Plots represent the following: A,E: % sLm invaded, B,F: % sLm phagocytosed, C,G: % of target cells invaded after 60 min, and D,H: the data from panels A-B compared in one plot for motile sLm-RT. Results are shown as mean ± SD for 20 independent simulations for motile sLm-RT (blue) and non-motile sLm-37 (gray). Vertical dotted lines indicate the default values of kattach = kφ = 0.051 s-1 used in the rest of the paper. (TIF) [file ppat.1011028.s030.tif]

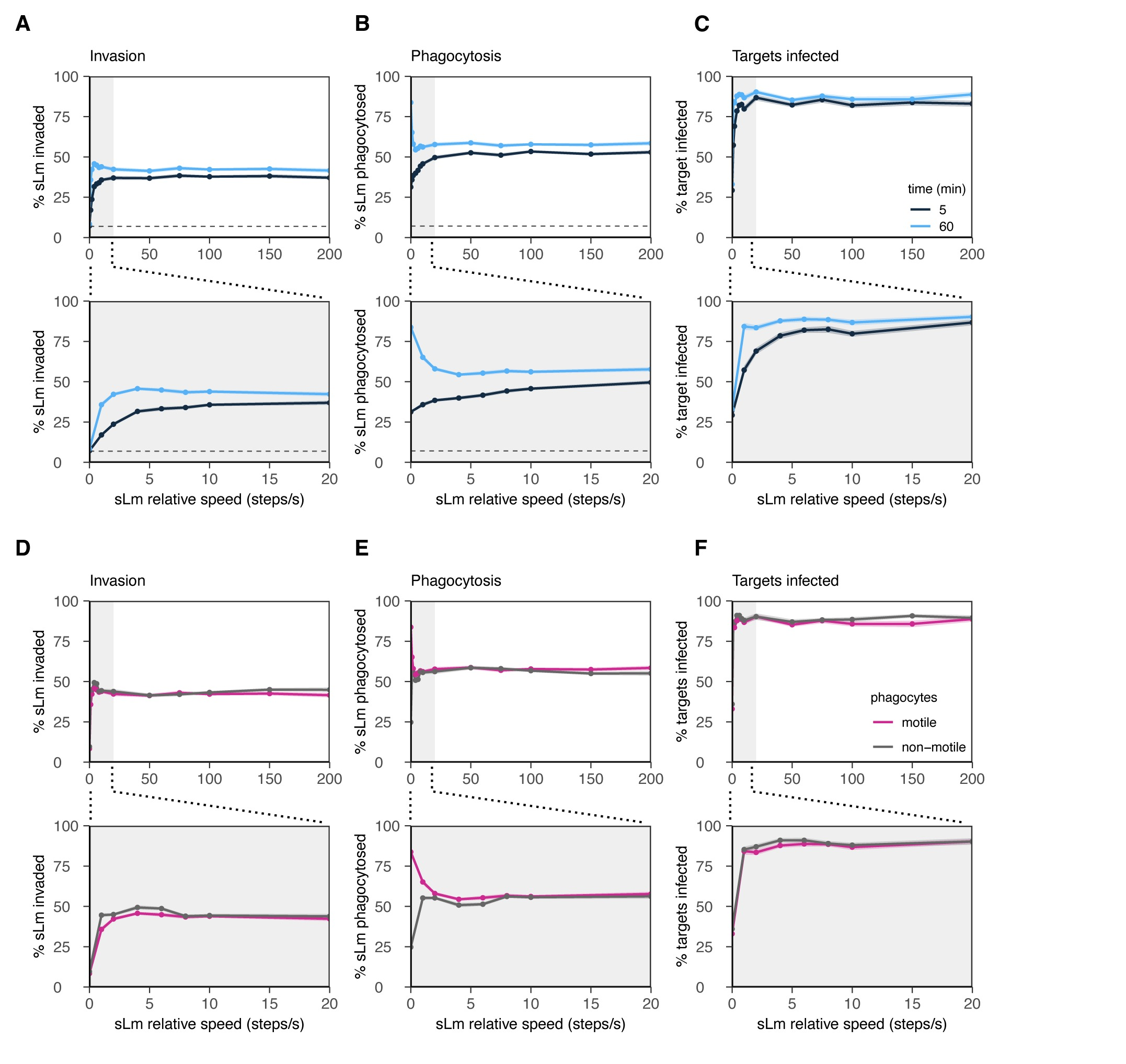

Supplement: S13 Fig — Immunological outcomes were assessed after varying A-C: the relative sLm speed in steps/s (see also S6 Fig), and D-F: phagocyte speed. Plots show A: % invaded sLm, B: % phagocytosed sLm and C: % target cells invaded after 5 or 60 min, with horizontal lines in A-B indicating the percentage of the surface area covered by target cells or phagocytes, respectively. The bottom plots in A-C are zoomed in on lower relative speeds, indicated by the gray shaded region in the top-row panels. D-F show only the 60-minute curve, but now for motile phagocytes (modelled with default parameters, pink) compared to non-motile phagocytes (modelled with λact = maxact = 0, gray). Phagocyte motility affects outcomes only in the (very) low range of sLm motility, where bacteria are static enough that phagocytes can actually “hunt” them. All lines represent mean ± SE of 20 independent simulations. (TIF) [file ppat.1011028.s031.tif]
