# Supplementary material for: Listeria motility increases the efficiency of epithelial invasion during intestinal infection
Source: PLoS Pathog. 2022 Dec 30;18(12):e1011028. doi: 10.1371/journal.ppat.1011028 (PMC9836302; doi:10.1371/journal.ppat.1011028)
Supplement: Attachment — Submitted filename: response to reviewers_11-14-2022.docx [file ppat.1011028.s032.docx]

Dear Reviewers, Dear Dr. Knodler,

We are grateful for the opportunity to submit a revised version of our attached manuscript for consideration at PloS Pathogens. We carefully revised the manuscript to address the issues raised in the manuscript critique (line numbers refer to the non-highlighted version of the manuscript). For details we refer to the point-by-point response below, but the most important changes are outlined here:

1. Two of the reviewers asked for further confirmation that Lm retains motility when traversing the digestive tract after oral infection (reviewer 1#2, reviewer 2#5). To address this, we have performed additional experiments to confirm that this is indeed the case. See: Movie S3, Supp. Fig. S3, section “Motile Lm arrive at the intestinal epithelium after oral infection in vivo” (lines 86-93)^[1]^ and discussion lines 312-325 of the revised manuscript.
2. Two of the reviewers also pointed out the possibility that other factors than motility could confound our comparisons between Lm-RT and Lm-37 (reviewer 1#1, reviewer 2#1 and 4). We have addressed this possibility in new experiments with a flaA mutant Lm (Supp. Fig. S4, Movies S8-S9, results lines 158-162). These experiments confirm that it is indeed motility per se that is responsible for the changes observed. We also added a discussion section (lines 355-382) to address this question.
3. Importantly, the reviewers questioned the relevance of simulating Lm-phagocyte interactions on the apical side of the epithelium (reviewer 1#3, reviewer 2#6). They rightfully questioned the kinetics of neutrophil recruitment and whether they would transmigrate to the apical side of the epithelium. We agree that the motivation for the simulations was unclear and thank the reviewers for pointing this out. We have now:
   - clarified in what context these interactions are expected to be relevant. While the reviewers rightfully point out that the recruitment of neutrophils takes several hours and would occur too late to prevent bacterial invasion after a single challenge, this is at least partly an artefact of in vivo models that infect mice in a single challenge. We argue that natural infections typically involve an ongoing bacterial challenge that continues for hours or days, leaving ample time for neutrophils to find their way to the epithelial surface and interact with Lm. We have carefully revised the text (abstract, introduction lines 44-46, and results lines 215-234) to better motivate these simulations.
   - performed additional experiments to confirm the relevance of this scenario. In a model of persistent infection, substantial numbers of neutrophils patrol the apical epithelium at 5hpi, where they indeed interact with Lm in the case of an ongoing infection (Supp. Fig. S8 and lines 215-234 of the revision).
4. Reviewer 2 asked for more evidence that bacteria are too fast for neutrophils to “hunt”, especially given established literature on the importance of neutrophil chemotaxis towards bacteria (reviewer 2#6). In response, we have investigated the dynamics of Lm-neutrophil interactions both in vitro and in an explant model (Fig. 5D,E, Movies S16,S17, results lines 295-302 and discussion lines 412-419). Excitingly, these results directly confirm what we observed in the model. We also note that this does not contradict established literature on the importance of neutrophil chemotaxis, which is still extremely relevant for many other bacteria that are not (as) motile as Lm. To avoid confusion, we have added a statement to this effect in the manuscript discussion (lines 432-435).

We thank the reviewers for their input, which we feel has considerably improved the manuscript. We have considered and addressed every point (see also point-by-point response below), and hope that with these revisions, our manuscript is now suitable for publication in Plos Pathogens.

With best regards, on behalf of all authors,
Mark Miller and Inge Wortel

**Point-by-point responses shown in blue**

**Reviewer #1 major issues:** 
1.1 In the first part of the study (Fig 1-3) the authors make the implicit assumption that only motility is relevant to consider when comparing how Lm-RT vs Lm-37˚C scout the epithelium and invade goblet cells. However, temperature shift will alter many aspects of virulence and it is known that the Lm master virulence regulator, PrfA, is under temperature control. To substantiate their claims, the authors should ensure that a flagellar mutant of Lm grown at RT behaves as the wild-type Lm grown at 37 ˚C with respect to invasion of Goblet cells (in connection to results presented in Fig 2-3).

See summary point 2, or below for a more detailed response:

We agree that temperature dependent changes in gene expression could also alter invasion and infection. However, Lm virulence factors are typically repressed at low temperatures and upregulated at 37 degrees (Leimeister-Wächter et al. 1990, Mengaud et al. 1991, Johansson et al., 2002). Thus, in our experiments, we would expect increased adhesion and invasiveness of Lm-37 relative to Lm-RT – yet we consistently see that Lm-RT invades more efficiently. Because our study focuses on the role of bacterial motility on target cell interactions, rather than downstream phenomena mediated by virulence factors such as LLO mediated phagosome escape or ActA mediated cell to cell spread, we believe comparing Lm-RT to Lm-37 is both physiological (mimics food borne infection) and conservative (i.e., would favor Lm-37 with increased virulence factor expression).

To further address this point, we obtained an flaA deletion mutant on the 10403 Lm strain (Gorsky et al., 2009, gift of Lisa Gorski, Ph.D., USDA). However, the flaA deletion mutant and parental strain are not “murinized” and as such are not appropriate for performing epithelial invasion assays in our mouse infection model system. Therefore, we performed imaging invasion experiments in human ileum obtained from the Wash U digestive disease research core (DDRCC). We first verified that the flaA deletion mutant was non-motile when grown at RT with bright field microscopy (Movie S8) and then assessed Lm epithelial interactions in the explant system using 2P microscopy (Movie S9, Supp. Fig. S4). In two independent experiments we found that the 10403 flaA deletion mutant had profound defects in motility, epithelial access, and adhesion, similar to 37°C cultured murinized 10403s in mouse and wild-type EGD in human ileum explants. These data have been added to the results (lines 158-162):

*“To confirm that this difference was due to Lm-RT motility, and not temperature-dependent changes in virulence factor expression, we performed 2P imaging experiments with a 10403 flaA deletion mutant (Gorski et al., 2009, gift of Lisa Gorski, USDA) grown at RT. Indeed, flagella-deficient Lm grown at RT had profound defects in motility, mucus penetration and epithelial adhesion, similar to those observed in murinized 10403s and wild-type EGD when cultured at 37 ˚C (Supp. Fig. S4, Movies S8,S9)”.*

We also added a discussion section (lines 355-382) on this topic. We hope that these data have addressed the reviewer’s concerns.

2. For the findings to be physiologically relevant, Lm-RT need to retain flagellar motility upon arrival in the intestinal lumen after a per-oral infection. Indeed, the authors show that Lm-RT switched to 37 ˚C in liquid broth retain motility for up to 3h (Fig S1), which is a good start. However, it should be straight-forward to conduct a per-oral infection in mice with labelled Lm-RT and assess to what extent motile Lm can be observed in gut luminal content at different time-points post-infection. This appears critical to support the main conclusion of the manuscript.

See summary point 1, or below for a more detailed response:

We agree this will be more compelling data. The main issue is that the binding of RT-Lm to the epithelium is very fast and efficient (essentially complete by 45 minutes in our explant system), leaving a very narrow “window of opportunity” when bacteria have reached a section of the intestine, but not yet invaded or bound to mucus or food particles and become non-motile.

To overcome these technical issues, we have infected mice orally (via gavage) with EGD-GFP Lm (which should not bind the epithelium efficiently via E-cadherin) and have observed motile bacteria as far down as the terminal ileum 1-1.5hpi. This data demonstrates that Lm motility persists in vivo after passing through the stomach and entering the small intestine. Moreover, motile bacteria reach the terminal ileum within 1-1.5hpi which is consistent with gut transit times in mice that have been measured using radiolabeling and fluorescent dextrans(Padmanabhan et al., 2013, Woting & Blaut, 2018). We have added these data (Movie S3, Supp. Fig. S3) and added lines 86-93 to the manuscript to discuss these findings.

3. The last section of the study, which simulates the presence of phagocytes atop the epithelial lining, includes a speculative leap. The authors state that it is unknown from what time-point of infection, and in what numbers, phagocytes arrive at the apical side of the epithelium. Knowledge of these basic parameters is key for the relevance of the simulations in Figure 5. Again, this could be addressed experimentally in a straight forward manner. In similar per-oral Lm infections as in my comment 2, fixed intestinal tissue could be cross-sectioned, stained for phagocyte and epithelial cell markers, and the number of phagocytes present at the apical side of the epithelium quantified across time-points. Based on experience from other gut infection models, I however predict that during the first hours of infection, where Lm may remain motile, close to zero transmigrated phagocytes will be observed. This is typically a later phenomenon coupled to the inflammatory response. Nevertheless, the temporal relationship between Lm motility in the lumen (comment 2) and arrival of phagocytes atop the epithelium needs to be sorted out.

See summary point 3, or below for a more detailed response:

We agree this is a major caveat to our modeling of bacterial/phagocyte interactions and that the motivation and context for these simulations was not clearly explained.

While others from Charles Parkos’ group have shown that neutrophils undergo transepithelial migration to the surface of the epithelium (Fleming et al., 2018, Azcuta et al., 2022, in press), our unpublished results indicate that neutrophil recruitment occurs 4-6hpi (intraluminal challenge). We have now added data to show that neutrophils are robustly recruited to the surface of the epithelium 5hpi as compared to sham treated mice (Supp. Fig. S8).

As pointed out by the reviewer, neutrophil recruitment kinetics suggest that they are not likely to be present on the epithelium in time to oppose initial invasion following a single oral challenge. However, while administering a single bolus of bacteria is used in many mouse infection models, this doesn’t closely mimic a natural or ongoing infection where pathogenic bacteria have contaminated a food or water source) and may pass through the gut for hours to days. Admittedly, even in persistent infection scenario we would not expect neutrophils to *prevent* initial infection based on the efficiency of Lm invasion, but we believe it is entirely plausible that neutrophils could limit further invasion events at the epithelium through phagocytosis.

To better contextualize the simulations, we also added new experiments to the manuscript (Supp. Fig. S8, Movie S17, Figure 5D,E) and simulated an ongoing infection by reinfecting the lumen of the ileum 5hpi (after neutrophils reach the ileal epithelium, Supp. Fig. S8) with dye-labeled Lm. We analyzed Lm and neutrophil interaction dynamics at two different time resolutions (24 or 16 seconds or 100msec) and calculated mean track speeds for phagocytes and bacteria. We found that contacts between phagocytes and Lm were overwhelmingly driven by bacterial motility, not phagocyte crawling, confirming predictions made by the L-CPM simulations (Figure 5D,E, Movie S17). This also demonstrates the utility of the modeling work for understanding the cellular dynamics that underlie host pathogen interactions.

We agree with the reviewer that this context of the phagocyte L-CPM simulations was unclear. We have now added a results section (lines 215-234) to incorporate these results and to clearly state that we are simulating an ongoing infection both with the rechallenge mouse infection model and the L-CPM phagocytes simulations.

**Reviewer #2: Major points:**

The authors rely on growth at 37 or 23 of one strain of Listeria and refer to studies in the literature which use mutants in bacterial motility with conflicting results, however they have generated a new model system in which they could and should test a bacterial chemotaxis or motility mutant to support their conclusions that bacteria are actively migrating towards goblet cells.

We agree this was confusing and have edited the text (line 96-97,109-111) to state clearly that we are not measuring or modeling bacterial chemotaxis in our study. Instead, our aim was to show that motility facilitates contacts with goblet cells - even when its direction is random and it is *not* specifically targeted at goblet cells. To further strengthen these findings, we also added results with a flaA motility mutant (Supp. Fig. S4, Movies S8, S9 and lines 158-162).

We agree with the reviewer that it would be interesting to determine whether Listeria is using chemotaxis to locate and invade goblet cells more efficiently than with random migration. This is a worthy topic for future studies and our imaging and computational models are well suited to address these issues, but we believe it is beyond the current scope of this manuscript.

1) In particular, they take videos with the bacteria migrating to “goblet cell” like structures but in the same video there are other green bacteria that have not been tracked or that do not move towards goblet cells. How were the bacteria with tracks selected for quantification? Are these data quantified? I mainly saw that they quantified the change in CFUs, which I think is extrapolated from imaging or conducted through separate experiments with mouse infection.

For these experiments, we focused our analysis on the tracks moving along the epithelium, excluding Lm moving freely in the lumen. We did this since this behavior was more relevant to our hypothesis that bacterial motility and epithelial scanning facilitates Lm-goblet cell interactions. Furthermore, imaging Lm at the epithelial surface has the important technical advantage that bacteria are confined in 3D and thus easier to track using 2PM despite their high motility. We have now explained this more clearly in the figure captions (Figures 2 and 3, Movie S5). Finally, we did not quantify the tracks in Figure 2 and 3 (which were just an illustration of scanning), but we did for the new data in Figure 5D,E, where we tracked many Lm in the vicinity of a neutrophils on the epithelium.

Importantly, we would like to stress again that we are not proposing that Lm move specifically towards goblet cells, but rather that robust random bacterial motility along the surface of the epithelium increases the likelihood that Lm will encounter their preferred invasion target cells – see also the previous point. This is why we quantified Lm binding near goblet cells in these figures, showing that Lm motility results in increased epithelial interactions and invasion. However, in the imaging experiments it is very difficult to separate the effects of increased penetration through the mucus layer versus increased scanning along the epithelium – which is part of the rationale for building the L-CPM model (Figure 4) which can address that question quantitatively. The adjusted text (lines 96-97,109-111) in the revised manuscript should now explain this more clearly.

2) If the authors want to say that the bacteria are moving towards goblet cells from the videos, the authors should confirm that these cells are indeed what they say that they are using a goblet cell marker such as MUC5A or another mucin. If they cannot do so with two-photon they could use fixed images from their explants. Similarly, using Rh-dextran as a marker of goblet cells later on would not differentiate between goblet cells and phagocytic cells (from the explant) that could also take up the dye. I assume that both mouse explants and human tissue explants could have associated phagocytic cells but please correct me if I am wrong.

We have now included immunofluorescence staining of human ileum and showed that Lm accumulate around large epithelial cells that are Rh-dextran and cytokeratin-18 positive (McDole et al., 2012, Knoop et al., 2015) (Figures S4).

3) For the human explants the authors could treat the tissue with gentamicin to assess internalized bacterial cells in addition to extrapolating CFUs based on GFP pixel intensity. They could also measure CFUs on the other side of the tissue if the bacteria can transcytose across the tissue layer. This has been done with mouse intestinal tissue before.

While we agree in principle that this experiment would strengthen the imaging results, technical issues in the explant system would likely make the data difficult to interpret. The human tissue is a biopsy specimen that has cut edges and the smooth muscle layer is removed. Because of this, Lm could bypass the epithelium and enter the underlying regions from the sides of the biopsy or regions where the barrier is damaged and disrupted due to preparation.

In fact, our imaging-based estimate of attachment and invasion was chosen specifically because it can be used at locations where the biopsy tissue and epithelial barrier are intact and thus provide a good test of Lm invasion efficiency. Moreover, our goal was to inform our L-CPM parameters and validate its predictions qualitatively in both human and mouse tissue rather than to quantify Lm CFU precisely.

4) The EGD strain of Listeria has a published mutation in PrfA, thus the virulence factors are constitutively produced in this strain. How is motility affected? Does PrfA negatively regulate motility? Could an abundance of virulence factors (InlA in particular) affect the conclusions of the study?

These are all excellent questions and are related to point 2 of the summary above. In addition, we have now included the following text to discuss these issues (lines 355-382).

*"Lm virulence genes are silenced at low temperatures but dramatically upregulated by PrfA expression at 37°C (Leimeister-Wächter et al. 1990, Mengaud et al. 1991). In groundbreaking work from the Cossart group, temperature was shown to regulate PrfA expression via changes in the mRNA secondary structure, which blocks ribosome entry at lower temperatures but allows PrfA expression at* *37°C to upregulate Lm virulence factors (**Johansson et al., 2002). This mechanism is separate from the downregulation of flaA expression at 37°C, which is mediated by the repressor MogR (**Grundling et al., 2004). A MogR deletion mutant was used to demonstrate that flaA downregulation is crucial for cell-cell spread and full virulence in vivo (Grundling et al., 2004).*

*Importantly, the EGD Lm strain used in this study has a mutation in PrfA, allowing it to express virulence factors constitutively independent of temperature – raising the question to what extent we can compare the RT and 37°C conditions in this strain. Nevertheless, we believe this comparison is valid for several reasons.*

*First, we found that the PrfA mutation did not negatively regulate EGD motility, which was equivalent to that of the murinized Lm 10403s strain without a PrfA mutation (in line with the idea that motility is regulated through a separate mechanism). Second, while the constitutive expression of virulence factors in EGD could still enhance the virulence of Lm-RT, we would expect a similar effect on Lm-37, suggesting that any differences between EGD RT and 37°C are independent of the PrfA mutation. Third, the virulence of different Lm strains has been evaluated carefully by Becavin et al., and the EGD PrfA mutation was found to affect host cell invasion at low temperatures (30°C) only but not in vivo (Becavin et al., 2014). Importantly, our experiments were performed under conditions (i.e., 37°C for ex plant imaging and in vivo CFU assays) where the PrfA mutation is not expected to affect the results or conclusions of our study. Fourth, our experiments with murinized Lm (10403s strain) clearly showed Lm-RT had an invasion advantage over Lm-37, mirroring the results with EGD and human ileum.”*

5) How long do flagella persist when switched to 37 across the various strains of bacteria? The authors mention that a certain percentage of bacteria still express flagella but if they collected CFUs shed from animals following digestion would the bacteria still be motile?

This is an important question, but it has never been studied in vivo to our knowledge; see also point 1 of the response summary above. In addition:

As for analyzing feces, we don’t anticipate that freshly shed bacteria would be motile due to the time spent at 37 degrees. One could also imagine that Lm in feces at RT for several hours might upregulate motility again. Unfortunately, this is not possible for us to assess with a CFU assay since it involves plating and culturing the bacteria. Thus, any changes in motility would be due to passage through the mouse, or due to the culture temperature during the CFU recovery.

Still, for the two strains examined in our study we found that they maintained motility for several hours, which is enough time for motile Lm to pass through the small intestine (Padmanabhan et al, 2013 and Woting & Blaut, 2018). Furthermore, we now provide data that 1-1.5hpi by oral gavage, motile Lm reaches the ileum and can engage in epithelial scanning behavior (Movie S3, Supp. Fig. S3, lines 86-93, lines 311-325).

6) While some neutrophils extravasate – adding a neutrophil layer on the apical side of the gut seems to confuse the message as the number of neutrophils patrolling would probably not reach that of the lumenal side of the epithelial layer – why did the authors not address the question of motility in co-culture in vitro of neutrophils and motile or non-motile bacteria without the epithelial layer?

See points 3 and 4 of the response summary on top of this document. Also:

We agree that it is unlikely that neutrophils would accumulate rapidly enough on the apical surface of the epithelium to oppose initial Lm invasion. In fact, this is a prediction that can be made from the rapid invasion kinetics observed with the L-CPM. However, our rationale is that an ongoing infection (lasting hours - days) would provide an opportunity for neutrophils and bacteria to overlap on the epithelial surface. We have more carefully explained this context throughout the manuscript, in particular in lines 215-234.

We have addressed this comment by imaging bacterial-phagocyte interactions 5hpi, when neutrophils are present on the (apical side of the) epithelium and showed that Lm and phagocytes on the epithelium do indeed interact. Moreover, bacterial speeds are ~50x faster than phagocytes, demonstrating that the relative differences in speed makes it impossible for phagocytes hunt motile bacteria (this is the same relative speed difference of a running human chasing a commercial jet at cruising speed). We have also imaged bacteria and neutrophils in vitro and confirmed that the capture of bacteria by phagocytes occurs not by neutrophil stalking but likely through a fly paper mechanism. See (Fig. 5D,E, Movies S16,S17, results lines 295-302 and discussion lines 412-419).

These conclusions of the model that bacterial motility facilitate phagocytosis are testable but I’m not sure that their model tests it under realistic conditions. It is hard to extrapolate this conclusion if phagocytes would not normally patrol the apical side of the layer. There is an entire microbiome that would be on that layer of cells, so maybe it would be more relevant to test bacterial density and its affect on Lm chemotaxing towards goblet cells rather than neutrophils which would not really be there in the density simulated.

These are all valid suggestions that could be the focus of future work. However, see our response above and point 3 of the response summary on top of this document: our rationale is that during an ongoing natural infection (rather than a single challenge typically used in controled lab experiments), neutrophils are likely present on the epithelium and could potentially interact with bacteria. Indeed, we have observed such interactions between neutrophils and Lm 5 hpi using a rechallenge approach (see response to the previous point and Figure 5D,E, Supp. Fig. S8, Movies S16,S17). Importantly, our modelling results were consistent even at phagocyte densities lower than those used in Figure 5 (see supplementary figure S11), so these model predictions would hold even if only few neutrophils transmigrate to the apical side of the layer.

We agree with the reviewer that this aspect of the manuscript was confusing, so we have updated the text to explain the rationale and context more clearly (throughout the text, but especially in lines 215-234).

There is also a very established literature of phagocytes chemotaxing towards bacteria and in vitro data showing that it occurs so overturning this literature by saying that “simulating bacterial and phagocyte dynamics to scale, our CPM clearly shows that neutrophils cannot possibly “hunt” motile bacteria – which move at their own speed” is difficult to believe when the neutrophils would not be hunting the motile bacteria, unless the motile bacteria retain their flagella on the lumenal side of the epithelium. The authors could easily test this finding by putting motile bacteria with neutrophils in vitro to see if the neutrophils are fast enough to catch the bacteria.

See point 4 of the response summary on top of this document. In response to this comment, we have now imaged phagocytes and bacteria both ex vivo and in vitro, and show that motile bacteria, which move 50-100x faster than migrating phagocytes, are too fast to be pursued by neutrophils. These results have been added to the revised manuscript (Fig. 5D,E, Movies S16,S17, results lines 295-302 and discussion lines 412-419).

To be clear, our statement is specific for the case of highly motile bacteria such as Lm. Indeed, there are many scenarios where bacteria are non-motile and adherent and in those cases chemotaxis towards bacteria *would* be important. We therefore do not see these results as contradicting the existing literature – rather, they reveal the impact of bacterial motility on phagocytosis at barrier surfaces and suggest that phagocyte chemotaxis is likely only relevant in the context of adherent bacteria. We have updated lines 433-435 in the manuscript to address this confusion.

**Reviewer #3 major issues:**

-Figure 2A-F. The images are very hard to interpret. A schematic showing orientation of the tissue and colour code for labels would help guide the reader. What is blue? The panel images indicate DAPI but the figure legend indicates autofluorescence.

We thank the reviewer for pointing this out. We have now included a schematic (Figure 2A) to provide the reader with an overview of the experimental approach and corrected the figure caption.

-Figure 2H. This data seems like it should be panel A. Also, were other tissues collected for CFU counts?

No other tissues were analyzed for this manuscript. The CFU experiment in spleen was performed to simply show that Lm-RT was more infectious in our system, consistent with what has been reported by O’Neil and Marquis, Infection and Immunity, 2006.

-Figure 3I. The green rings of Lm observed are fascinating. The authors presume that Lm is binding to E-cadherin on goblet cells. However, Figure 3C shows that E-cadherin does not have a strong signal around the periphery of what are presumed goblet cells. Is this just a matter of the image chosen? Or are the rings created by Lm binding to themselves as well as the host cell?

Thank you for pointing this out. The binding of Lm to E-cadherin has been well established by Marc Lecuit’s group in mouse and human tissue systems (Lecuit et al., 1999, Nikitas et al., 2011, Kim et al., 2021). However, Lm tropism for goblet cells is believed to be due to E-cadherin being more accessible at the apical surface of goblet cells as compared to other epithelial cell populations. We also agree that Lm-Lm interactions could stabilize the ring structures, and this is an interesting avenue for future study. Indeed, there are multiple examples of Lm forming clumps in the mucus layer or rings around detached epithelial cells.

**Part III – Minor Issues: Editorial and Data Presentation Modifications**

Reviewer #1: 4. Abstract, Author summary, beginning of Discussion et.c.: Here, the authors make statements like “little is known regarding the effect of bacterial motility on invasion…”. This may be true for Lm, but not for gut bacterial infection in general. In particular for Salmonella, a string of published papers has detailed how flagellar motility enables the pathogen to find mucus gaps, efficiently reach the epithelial surface, and scout it by near-surface swimming to locate ideal invasion sites. The authors are advised to rephrase these passages to better reflect the current knowledge front.

Thank you for catching this oversight. We have now rephrased these statements and added several citations for the role of Salmonella motility in invasion (lines 31-32, 326-354) and believe this strengthens the overall message of the study. We have also changed the manuscript title slightly from “Listeria motility increases the efficiency of goblet cell invasion during intestinal infection) to: Listeria motility increases the efficiency of epithelial invasion during intestinal infection”, which we feel encompasses the finding that in addition to goblet cell interactions, Lm motility also facilitates access to the epithelium similar to what has been reported to for Salmonella infection.

We have now added the following text to the discussion (lines 326-354):

*“The role of flagellar motility has been extensively investigated in the context of Salmonella pathogenesis. In an oral infection model (newly hatched chicks), non-flagellated mutant Salmonella was found to be less virulent in vivo, but using a gut explant approach, the authors concluded that this was likely due to flagella mediating epithelial adhesion rather than motility per se (Allen-Vercoe et al., 1999, Allen-Vercoe and Woodward, 1999). In contrast, when the same Salmonella mutant strain was examined in a rat ileal explant model, motility enhanced infection, presumably by increasing bacterial interactions with the intestinal epithelium (Robertson et al., 2000). Likewise, Barbosa et al. found that chicks infected by gavage with non-motile (ΔmotB) and non-flagellated (ΔfliC ) Salmonella generated significantly lower CFU 3-5 dpi in the cecum compared to wild-type Salmonella Enteritidis (Barbosa et al., 2017). The importance of motility to Salmonella infection was further elucidated in a fascinating study by Furter et al, who demonstrated with live microscopy of explanted mouse colon and cecum that motility allowed Salmonella to probe the mucus barrier for defects and access the epithelium (Furter et al, 2019). […] In our current study, we used a combination of different model systems to show that motile Lm more readily penetrates the mucus barrier above villi and gains access to the epithelial surface of the ileum. This finding resembles the results in the Salmonella model (Furter et al, 2019), although the explanted ileum has a less dense mucus layer compared to the colon studied there. Indeed, future iterations of the L-CPM model could incorporate a simulated mucus layer to investigate how motile bacteria interact with the mucus, which is relevant to understanding bacterial pathogenesis as well as the colonization of healthy microbiota.*

*5. Page 3, line 13: “One example is the forming protein”. The word “pore” is missing.*

Thank you. This has been corrected.

6. Page 3, line 31 and elsewhere: The authors talk about “immunological outcomes” at many places in the text. However, this manuscript completely lacks data on immunological outcomes (i.e. changes in histopathology, induction of cytokine production, immune cell recruitment into the mucosa, antibody levels in blood or similar). Hence, another wording would be more appropriate.

We have now edited the text throughout the manuscript to refer only to binding and CFU as “outcomes” or a measure of target cell engagement and invasion.

7. Page 4, line 64-65: The % motile Lm under these different conditions is an important metric, but is hidden in Fig S1. Please clarify.

This is now discussed more prominently (lines 82-84, in addition to the new section in lines 86-93).

8. Figure 2-3 legends and results text: The reader has to go to the Methods to figure out which gut segment has been used. Please clarify.

Thank you for pointing this out. We have now corrected the legends and text throughout the manuscript to specify that the experiments were conducted in the ileum.

9. Legends to Figure S6 and Supplementary Movies S1-S6, S8: These lack information on scale bars, color schemes, time stamps et.c. Please amend.

Again, thank you. We have now added this information directly to the supplemental movie legends.

Reviewer #2: (No Response)

Reviewer #3:

-line 83, “Sections of B6 mouse intestine were harvested, glued to plastic…”. A schematic would help guide the reader as to how tissues were processed, and infections performed.

In response to this comment, we have now added a schematic (figure 2A) to better explain the experimental system.

-line 264 …”Lm-RT (both EDG and 10403S strains)”… should be EGD

Thank you, this has been corrected.

1. Line numbers refer to the revised manuscript file, not the version with changes highlighted.
